# The Dutch Solid Start program: describing the implementation and experiences of the program's first thousand days

**DOI:** 10.1186/s12913-023-09873-y

**Published:** 2023-08-30

**Authors:** Joyce M. Molenaar, Inge C. Boesveld, Jeroen N. Struijs, Jessica C. Kiefte- de Jong

**Affiliations:** 1 Department of Quality of Care and Health Economics, Centre for Nutrition, Prevention and Health Services, National Institute for Public Health and the Environment (RIVM), 3721 MA Bilthoven, the Netherlands; 2 Department of Public Health and Primary Care/ Health Campus The Hague, Leiden University Medical Centre, 2511 DP The Hague, the Netherlands

**Keywords:** Cross-sectoral collaboration, Integrated care, Health inequities, Maternal and child health, First thousand days, Solid Start program, Rainbow Model of Integrated Care

## Abstract

**Background:**

In 2018, the Dutch government initiated the Solid Start program to provide each child the best start in life. The program focuses on the crucial first thousand days of life, which span from preconception to a child’s second birthday, and has a specific focus towards (future) parents and young children in vulnerable situations. A key program element is improving collaboration between the medical and social sector by creating Solid Start coalitions. This study aimed to describe the implementation of the Dutch Solid Start program, in order to learn for future practice and policy. Specifically, this paper describes to what extent Solid Start is implemented within municipalities and outlines stakeholders’ experiences with the implementation of Solid Start and the associated cross-sectoral collaboration.

**Methods:**

Quantitative and qualitative data were collected from 2019 until 2021. Questionnaires were sent to all 352 Dutch municipalities and analyzed using descriptive statistics. Qualitative data were obtained through focus group discussions(*n* = 6) and semi-structured interviews(*n* = 19) with representatives of care and support organizations, knowledge institutes and professional associations, Solid Start project leaders, advisors, municipal officials, researchers, clients and experts-by-experience. Qualitative data were analyzed using the Rainbow Model of Integrated Care.

**Results:**

Findings indicated progress in the development of Solid Start coalitions(*n* = 40 in 2019, *n* = 140 in 2021), and an increase in cross-sectoral collaboration. According to the stakeholders, initiating Solid Start increased the sense of urgency concerning the importance of the first thousand days and stimulated professionals from various backgrounds to get to know each other, resulting in more collaborative agreements on cross-sectoral care provision. Important elements mentioned for effective collaboration within coalitions were an active coordinator as driving force, and a shared societal goal. However, stakeholders experienced that Solid Start is not yet fully incorporated into all professionals’ everyday practice. Most common barriers for collaboration related to systemic integration at macro-level, including limited resources and collaboration-inhibiting regulations. Stakeholders emphasized the importance of ensuring Solid Start and mentioned various needs, including sustainable funding, supportive regulations, responsiveness to stakeholders’ needs, ongoing knowledge development, and client involvement.

**Conclusion:**

Solid Start, as a national program with strong local focus, has led to various incremental changes that supported cross-sectoral collaboration to improve care during the first thousand days, without major transformations of systemic structures. However, to ensure the program’s sustainability, needs such as sustainable funding should be addressed.

**Supplementary Information:**

The online version contains supplementary material available at 10.1186/s12913-023-09873-y.

## Background

Preconception, pregnancy and the first two years of life (the first thousand days) are crucial for children’s development and health, and a decisive period in the emergence of health inequities [1, 2]. These avoidable differences in health outcomes [3] that start in early life pose an important challenge [2]. Years of research that aimed to understand the nature and scope of health inequities showed both social and medical-related drivers, hence they should be addressed together in reducing health inequities [2, 4–6]. Factors such as poverty, housing difficulties, stress and unemployment also highly influence health and wellbeing and cannot be addressed in the medical sector alone. Therefore, as stressed in several recent studies and reports, cross-sectoral collaboration between actors from the medical, social and public health sectors is considered essential to provide every child the best start in life [2, 7–10].

Internationally, multiple countries have implemented programs and policy reforms to reduce health inequities by integrating medical and social services in early life [11–14]. In the Netherlands, the nationwide action-program ‘Solid Start’ (in Dutch: Kansrijke Start) was launched by the Dutch Ministry of Health, Welfare and Sport (Dutch abbreviation: VWS) in 2018 [15]. The program aims to provide each child the best start in life by stimulating cross-sectoral collaboration, with a specific focus towards (future) parents and young children in vulnerable situations. The program strategy is based on the foundations of previous programs that aimed to integrate medical and social services, including the local ‘Ready for a baby’ program in Rotterdam (2008–2012) [16] and the subsequent ‘Healthy Pregnancy 4-All’ programs in several municipalities (since 2011) [7, 17, 18]. Solid Start has a comprehensive population-based and upstream strategy, which means that its preventive and supportive measures aim to address the underlying factors that influence health and wellbeing at an early stage, in order to prevent or mitigate problems in later life. Policy measures were implemented for three periods: prior to pregnancy, during pregnancy and after birth, in order to prevent inequity and improve later health and well-being. The measures are aimed at preventing unintended pregnancies, preparing parents better for pregnancy, identifying medical and non-medical problems sooner, and supporting (future) parents in vulnerable situations better. The Dutch government financially supported municipalities to build a cross-sectoral approach for the first thousand days by forming or strengthening integrated ‘Solid Start coalitions’. These coalitions consist of representatives of local organizations and providers working in the medical, social and public health domain, including midwives, obstetricians, maternity care assistants, youth healthcare providers, neighborhood/social teams, social workers, debt counselors, and municipal officials. The approach is supposed to be based on local data, challenges and existing networks. Hence, each municipality formulates its own objectives, agreements, actions and strategy to tackle the local problems.

Previous studies on collaboration during the first thousand days often focused on either the medical or social sector, or a specific temporal window such as pregnancy or after birth only. For example, several studies within the medical sector in the Netherlands [19–23] and in other countries [24–26] reported on facilitators and challenges with interprofessional and interorganizational collaboration during pregnancy and childbirth. Some of the reported challenges were competition, suboptimal communication, power imbalances and fragmented structures, while facilitators included trust, feeling valued, formalized procedures and insight into each other's knowledge and competences [19–22, 24–26]. Other studies that reported on integrated youth (health)care [27–29] found similar facilitators and challenges and also mentioned the need for further collaboration. Collaboration in maternity care is often described as complex and not self-evident, as healthcare providers historically have worked relatively autonomous with separated organizational structures, education programs, protocols, cultures and practices [8, 22, 30]. More integrated care requires changes at different interrelated levels (micro, meso and macro), as outlined by Valentijn and colleagues [31].

Although these previous studies have furthered our understanding on collaboration, to date, there is limited knowledge into the development of cross-sectoral collaboration between the medical and social sector during the complete trajectory of the first thousand days as only few studies have devoted attention to this topic as a whole [7, 8]. This knowledge is particularly relevant as we do not know if collaboration between sectors presents different challenges compared to collaboration within a sector, due to for example the larger differences in cultures and structures. Moreover, limited qualitative research has been conducted to comprehensively examine client experiences within the Dutch context [32], despite enhanced client experiences being one of the ultimate goals of cross-sectoral collaboration and integration. Existing studies primarily include either the perspectives of healthcare professionals and policymakers, or adopt a more quantitative approach [33, 34]. The overall exploration of the implementation of Solid Start can be enriched if the viewpoints of those who provide, organize, examine ánd receive care are considered. Additionally, monitoring and reflecting on the development towards cross-sectoral collaboration during the implementation of a national policy program is important to support learning for future practice and policy developments in this direction.

Therefore, in this study, we aimed to describe the implementation of the Dutch Solid Start program during 2019, 2020 and 2021. We formulated the following two research questions: 1) To what extent is the Solid Start program implemented within municipalities? 2) What are the experiences of stakeholders with the implementation of the Solid Start program and cross-sectoral collaboration?

## Methods

### Research design

**Fig. 1:**
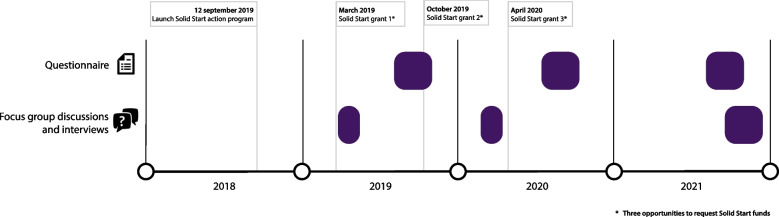
Timeline data-collection

The first research question was answered by using quantitative data from questionnaires among municipalities. The second research question was answered with qualitative data from interviews and focus group discussions (FGDs). We had several rounds of data collection in subsequent years after the implementation of the nationwide Solid Start program in September 2018 (Fig. 1).

### Study setting

The national Solid Start program was launched by the Dutch government in September 2018. The Ministry of VWS facilitated various (supportive) measures, including the possibility for municipalities to request Solid Start funds at three time points (March 2019, October 2019 and April 2020) to start building or strengthening their local Solid Start coalition. The funds could be utilized at municipality level to start a local coalition, but municipalities could also choose to pool their resources and collectively work towards a (sub-)regional approach or coalition with other municipalities. Municipalities were in the lead to create coalitions of partners from the medical and social sector who jointly made agreements about care and support during the first thousand days and to families in vulnerable situations. Some basic elements of these coalitions were set [35]. Municipalities received support from Pharos (the Dutch Centre of Expertise on Health Disparities) to build their coalition. Additionally, municipalities were able to use an analysis tool to map the current and envisioned situation, an inventory of effective interventions, key local, regional and national data, and inspiration from successful examples across the country.

Appendix 1 provides a description of the Dutch care and support system during the first thousand days. This study was part of the national monitor of the Solid Start program that is conducted by the National Institute for Public Health and the Environment (Dutch abbreviation: RIVM) by commission of the Ministry of VWS. The RIVM monitors the implementation of the Solid Start program by collecting both quantitative data on process- and outcome indicators as well as qualitative data on experiences and developments. Since 2021, the RIVM also provides support to municipalities in monitoring their local approach. Appendix 2 provides an overview of the national and local monitor.

### Quantitative data collection

#### Participants

The questionnaire was distributed among all municipalities that requested funds in 2019 (*N* = 147) and among all municipalities -including those without funds- in 2020 and 2021 (*N* = 355 and 352 respectively). The Ministry of VWS invited the municipalities that requested funds to participate in the questionnaire, the Association of Dutch Municipalities (Dutch abbreviation: VNG) invited the other municipalities to participate in the questionnaire.

#### Data collection

The online questionnaire focused on the local implementation of Solid Start and consisted of questions about municipalities’ development towards Solid Start coalitions. The questions mainly had closed answer categories and were slightly different each year depending on national developments and new insights. The questions that were relevant to this article and comparable over the years included the following topics: Solid Start funds, local coalition, action plan, goals and ambitions, partners, activities, monitoring, support and COVID-19. Examples of questions included: ‘Has your municipality formed a Solid Start coalition?’ and ‘What is the status of monitoring Solid Start in your municipality?’ An overview of the questions can be found in Table 2 (results section).

#### Data analysis

Quantitative data were analyzed using descriptive statistics. We used Excel and R to calculate frequencies and percentages.

### Qualitative data collection

#### Participants

For the interviews and FGDs, we used purposive sampling to ensure heterogeneity [36]. We invited representatives of care and support organizations (managers and care providers), Solid Start project leaders or advisors, other municipal officials, representatives of national knowledge institutes and professional associations, and researchers to join a FGD at a predefined time. In 2021, we organized individual interviews with those not available if their perspective was otherwise missing. Additionally in 2021, we invited clients and experts-by-experience for individual interviews at their preferred time and place, because we wanted to create the conditions in which they felt comfortable to share their personal stories in more detail than possible during a FGD. Clients received care and support during the first thousand days at the time of the interview. The experts-by-experience had collective experiential knowledge or were trained in using personal and collective experiences to support families in vulnerable situations. Most participants received an invitation to participate directly through an e-mail by the research team. One of the experts-by-experience supported the recruitment of clients by providing them information and discussing a feasible date and place.

#### Data collection

The qualitative data were collected online (2020 and 2021, as a consequence of COVID-19 regulations) or live (2019 and several interviews in 2021). The interview guide focused on the experiences with the implementation of the Dutch Solid Start program and included a series of fixed open questions that were similar in each interview or FGD, and flexible questions adapted to the type of respondents or year of data-collection to reflect the progress of the Solid Start program. Table 1 provides an overview of the main topics. FGDs lasted between 70 to 110 min. Interviews lasted on average 35 min, ranging from 11 to 52 min. All individual interviews were held one-on-one, with some exceptions. The expert by experience who assisted with client recruitment was also present during these interviews with clients to provide reassurance to clients and ask supplementary questions to gain more meaningful insights. Additionally, 4 project leaders and advisors within the same coalition were interviewed together.

**Table 1:** Topics in FGD’s and interviews

**General topics**		
• General experiences with Solid Start within the organization/ municipality/ region • Involved parties • Collaboration between medical and social sector (in the formation of coalitions and in daily practice) • Facilitators: what went well, factors that facilitated development • Barriers: what went wrong, factors that impeded development • Needs for the future and priorities		
**Year-specific topics**		
*2019 (shortly after the start of the program in sept. 2018)* • Transition: before and after implementation of Solid Start • Relation between previous/ current initiatives and Solid Start	*2020* • Funding and financing • Objectives and monitoring • Knowledge exchange	*2021 (shortly before the end of the initial program)* • Effects/ added value of Solid Start • Continuity of the program • Involvement of experts-by-experience • Early detection (screening) • Support for professionals • Solid Start as example for other sectors?

#### Data analysis

**Fig. 2:**
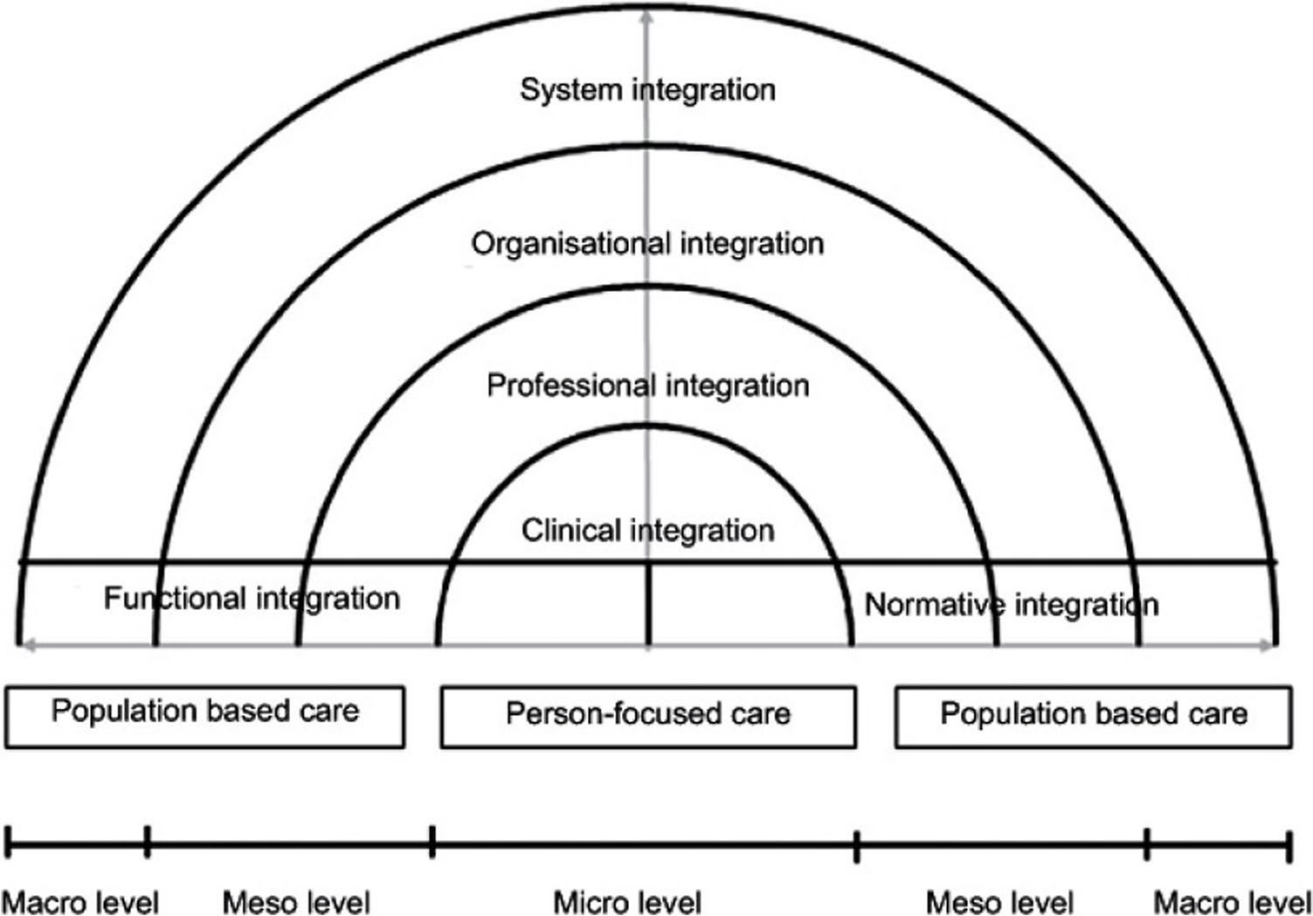
Rainbow model of integrated care (RMIC) by Valentijn et al. (2013)

All interviews and FGDs were audio-recorded, transcribed verbatim and analyzed in MaxQDA. We conducted a thematic analysis based on deductive coding, while remaining open to add relevant elements emerging from the data. A coding frame was set based on the Rainbow Model of Integrated Care (RMIC) by Valentijn et al. (2013). The RMIC was developed as a framework to describe integrated care in 6 interrelated dimensions (Fig. 2). Integrated care, in our paper, refers to the collaborative efforts of multiple professionals and organizations across the medical and social care system to provide comprehensive, accessible, and coordinated care for the benefit of (future) parents and their children [37, 38]. The RMIC outlines contact between client and care provider at microlevel (clinical integration), collaboration between professionals and organizations at mesolevel (professional- and organizational integration) and the wider policies and rules within the health system that influence collaboration at macrolevel (system integration). These levels are linked and enabled through supportive structural functions such as resources- and information management (functional integration) and softer aspects including shared vision, culture and informal collaboration (normative integration). The six dimensions are outlined in a taxonomy of 59 key features [38]. We used these 59 key features for coding and described the results according to the 6 dimensions. Two authors (JM and IB) coded the first 2 transcripts independently and compared coding to refine the coding frame. Next, JM coded all transcripts and IB cross-checked coding for three transcripts. The codes were analyzed and discussed in several meetings with the research team. Doing so, we sought for links between levels of integration within the RMIC and for patterns over the years.

## Results

The results are presented in two sections according to the research questions. In part one, we explain to what extent the Solid Start program is implemented within municipalities. In part two, we outline the experiences of stakeholders with the implementation of the Solid Start program and cross-sectoral collaboration.

### Development towards Solid Start coalitions

**Fig. 3:**
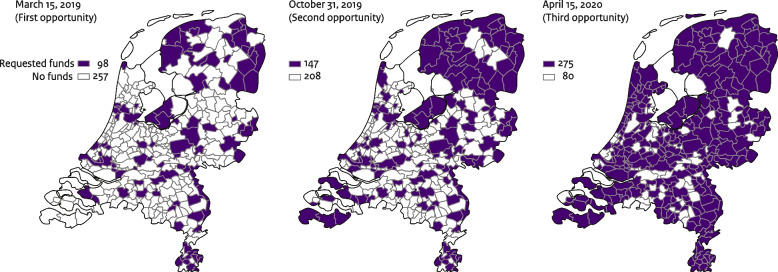
Municipalities that requested the Solid Start funds

There were 355 municipalities in the Netherlands in 2019 and 2020, whereas there were 352 in 2021 due to merging. Municipalities had the opportunity to request the Solid Start funds from the Dutch government at three time points: March 2019, October 2019 and April 2020. The first two rounds were only open to a specific group of 150 municipalities that joined the national Health In The City program (in Dutch: Gezond In De Stad), focused on tackling health inequalities at local level. The number of municipalities that requested funds increased from 98 in March 2019 to 275 in April 2020 (Fig. 3).

#### Solid Start coalitions

**Fig. 4:**
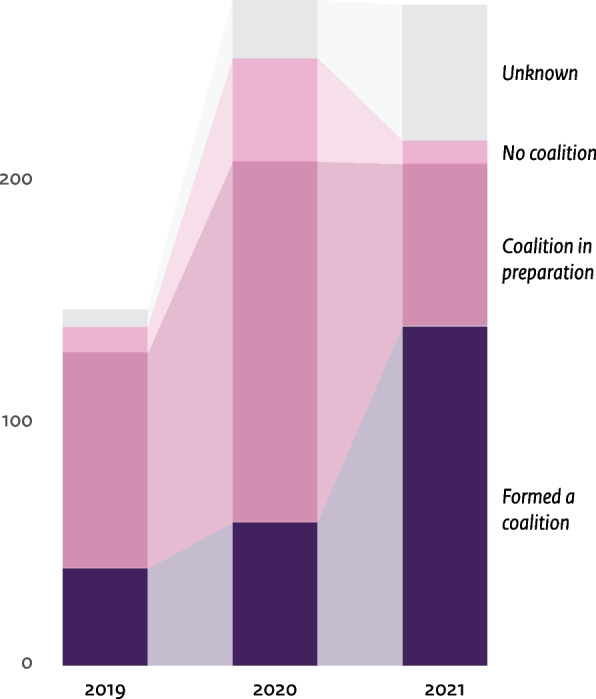
Development of coalitions during 2019 – 2021 The figure shows municipalities' answers to the question 'Did you form a local Solid Start coalition?'

Municipalities completed an online questionnaire in 2019 (*n* = 140), 2020 (*n* = 251) and 2021 (*n* = 217) (Table 2). Figure 4 shows the number of municipalities reporting to have formed a local coalition across the country. The numbers increased since 2019 (*n* = 40), especially from 2020 (*n* = 59) to 2021 (*n* = 140). Around half of the municipalities that had a coalition in development in 2020, reported to have formed their coalition a year later. In 2021, 65% (*n* = 140) of the responding municipalities that received funding formed a coalition. More than half of them collaborated with other municipalities (Table 2).

**Table 2:** Overview of quantitative findings

		**2019**	**2020**	**2021**
**Number of municipalities in the Netherlands**		355	355	352^d^
	Number of municipalities that requested the Solid Start funds	147	275	273^d^
	Number of municipalities that have been invited to participate in the questionnaire	150^b^	355	352^d^
**Number of municipalities that responded to the questionnaire**		140	289	229
	Response among municipalities with the Solid Start funds	140 (95%)	251 (91%)	217 (79%)
	Response among municipalities without the Solid Start funds	Not applicable	38 (48%)	12 (15%)
**Topics**	**Questions + answer categories**	**n (% among all municipalities with Solid Start funds, % among all municipalities **^c^**)**		
**Coalition**	**Has your municipality formed a Solid Start coalition?**			
	Yes	40 (27%, 11%)	59 (21%, 17%)	140 (51%, 40%)
	No, not yet. We are in talks/ preparation for forming a coalition	89 (61%, 25%)	149 (54%, 42%)	67 (25%, 19%)
	No	11 (7%, 3%)	43 (16%, 12%)	10 (4%, 3%)
	**Is your municipality collaborating with other municipalities to form or strengthen a (sub-)regional coalition?**			
	Yes	No data	157 (57%, 44%)	138 (51%, 39%)
	No	No data	94 (34%, 26%)	79 (29%, 22%)
**Topics**	**Questions + answer categories**	**n (% among all municipalities responding to the question, % among all municipalities **^c^**)**		
**Plan of action**	**Has your municipality developed an action plan or roadmap together with collaborative partners for Solid Start?**			
	We have an action plan	26 (19%, 7%)	39 (14%, 11%)	73 (32%, 21%)
	We have started an action plan	48 (34%, 14%)	100 (35%, 28%)	83 (36%, 23%)
	No	23 (16%, 6%)	148 (52%, 42%)	72 (32%, 20%)
	Other	43 (31%, 12%)	-	-
**Objectives and activities**	**Has any objectives been set for Solid Start within your coalition/municipality?**^a^			
	Yes	No data	44 (15%, 12%)	100 (44%, 28%)
	In development	No data	155 (54%, 44%)	92 (41%, 26%)
	No	No data	87 (30%, 25%)	29 (13%, 8%)
	Other	No data	17 (6%, 5%)	5 (2%, 1%)
	- **If yes/ in development/ other, what time period(s) are the objectives focused on?**^a^			
	- Before pregnancy	No data	171 (81%, 48%)	156 (79%, 44%)
	- During pregnancy	No data	200 (95%, 56%)	185 (94%, 53%)
	- After birth till the age of 2	No data	195 (92%, 55%)	184 (93%, 52%)
	- After birth till the age of 4 or 18	No data	77 (36%, 22%)	60 (30%, 17%)
	**Have joint ambitions around Solid Start been formulated within your municipality/coalition?**			
	Yes	68 (49%, 19%)	122 (43%, 34%)	166 (72%, 66%)
	No	72 (51%, 20%)	165 (57%, 46%)	63 (28%, 25%)
	**Is Solid Start part of a wider policy framework?**			
	Yes	No data	250 (87%, 70%)	196 (86%, 56%)
	No	No data	36 (13%, 10%)	32 (14%, 9%)
	**Have collaborative agreements been made on an approach to the first thousand days and families in vulnerable situations?**^a^			
	Yes, at the implementation level	54 (39%, 15%)	95 (33%, 27%)	137 (60%, 39%)
	Yes, at the managerial/policy level	19 (14%, 5%)	44 (15%, 12%)	60 (26%, 17%)
	No	85 (61%, 24%)	171 (60%, 48%)	82 (36%, 23%)
	**Are there any activities within your municipality on the topic of Solid Start?**			
	Yes	No data	198 (70%, 56%)	182 (80%, 51%)
	No	No data	86 (30%, 24%)	46 (20%, 13%)
	- **If yes, when did these activities begin?**			
	- After the implementation of the Solid Start program (September 2018 – present)	No data	101 (53%, 28%)	122 (67%, 35%)
	- Before the implementation of the Solid Start program (before September 2018)	No data	89 (47%)	60 (33%, 17%)
**Monitoring**	**What is the status of monitoring Solid Start in your municipality?**^a^			
	We monitor the progress (process) of our program (e.g. implementation of interventions)	No data	59 (21%, 17%)	71 (31%, 20%)
	We monitor the outcomes of our program (e.g. pregnancy outcomes)	No data	33 (12%, 9%)	22 (10%, 6%)
	We have plans to monitor progress	No data	105 (37%, 30%)	102 (45%, 29%)
	We have plans to monitor outcomes	No data	81 (29%, 23%)	75 (33%, 21%)
	None of the above	No data	112 (39%, 32%)	43 (19%, 12%)
	**Do you have insight in the statistics and facts concerning the first thousand days in your municipality? (2019)/ Did you conduct a baseline assessment to gain insight in the statistics and facts concerning the first thousand days in your municipality (2020 and 2021)**			
	Yes	102 (73%, 29%)	118 (42%, 33%)	154 (68%, 44%)
	No	38 (27%, 11%)	166 (58%, 47%)	71 (32%, 20%)
**COVID-19**	**Has the COVID-19 pandemic affected activities and progress of Solid Start in your municipality/coalition?**^a^			
	Yes, it has caused a delay	No data	197 (70%, 55%)	167 (75%, 47%)
	Yes, other (e.g. changes in the approach/ strengthening of collaboration/ fewer financial resources for Solid Start)	No data	8 (3%)	16 (7%, 5%)
	No, it has not had any major consequences so far	No data	68 (24%, 19%)	52 (23%, 15%)
**Partners**	**Which parties are part of the local coalition or with which parties do you collaborate?**^a^** (see **Fig. 5** for an overview of specific partners in 2021)**			
	At least 1 partner in the medical sector	110 (79%, 31%)	225 (78%, 63%)	217 (95%, 86%)
	At least 1 partner in the social sector	120 (86%, 34%)	262 (91%, 74%)	224 (98%, 89%)
	At least 1 partner within the municipal organization (other departments)	96 (69%, 27%)	255 (89%, 72%)	220 (96%, 87%)
	At least 1 partner within the community	No data	No data	83 (36%, 33%)
	**Which parties that are not currently involved would you like to consult with? (top 3)**^a^			
	General practitioners	No data	No data	77 (34%, 22%)
	Experts-by-experience	No data	No data	55 (20%, 16%)
	Health insurers	No data	No data	44 (16%, 13%)

^a^Multiple answers allowed

^b^In 2019, only Health In The City municipalities (In Dutch: Gezond In De Stad (GIDS) gemeenten) were requested to participate (n = 150). At that time, they were the only municipalities that could request the Solid Start funds

^c^The percentage in relation to the total number of municipalities is often an underestimation, given the large number of missing values, particularly for municipalities that did not apply for the Solid Start funds. All percentages can be considered conservative, representing a minimum lower limit; the actual percentage is likely to be higher

^d^The number of municipalities changed during the years due to the merging of municipalities

#### Development within municipalities

Over the years, the number of municipalities with a plan of action, objectives, ambitions and activities increased (Table 2). By 2021, almost all responding municipalities (85%) were engaged in setting objectives. More than one in four municipalities set objectives aimed at a longer period (children until 4 or 18 years) than the original Solid Start program (up to 2 years), and Solid Start was almost always part of a wider policy framework. In 2021, 64% of the responding municipalities made collaborative agreements about the Solid Start approach at implementation level, managerial/policy-level, or both. Moreover, 80% of the responding municipalities reported having activities on the topic of Solid Start, and two-thirds of them started these activities in the timeframe after receiving the Solid Start funds. The quantitative data also showed that several municipalities started with monitoring Solid Start, and many reported having plans to monitor. Municipalities reported that they more often monitored processes than outcomes. Additionally, 68% of the responding municipalities in 2021 conducted a baseline assessment to gain insight into the statistics and facts concerning the first thousand days in their municipality. Three-quarters of the municipalities indicated that COVID-19 influenced Solid Start activities and progress in 2020 and 2021; it mostly caused a delay.

#### Involved stakeholders

**Fig. 5:**
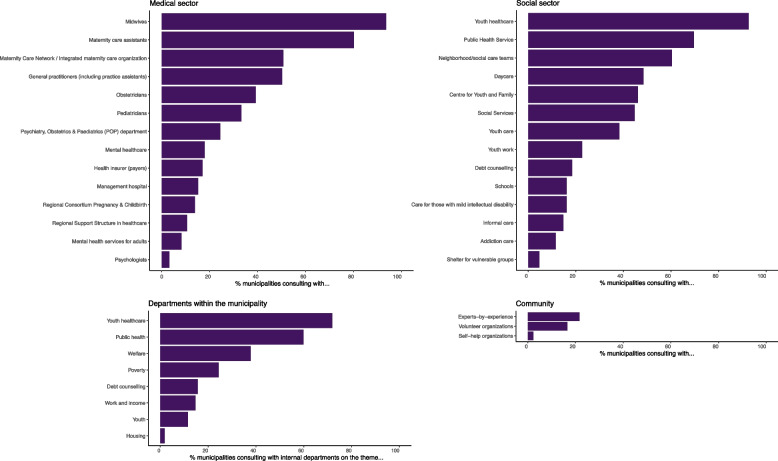
Parties that are part of the local coalition or with whom municipalities collaborate

There was a wide variety of stakeholders involved in Solid Start. Figure 5 shows which parties municipalities mentioned when they were asked who is part of the local coalition or with whom they collaborate. Most often mentioned were midwives, maternity care assistants, youth healthcare, Public Health Services, neighborhood/social teams and policy makers within other municipal departments on the topics of youth healthcare and public health. In 2021, around one-third of the municipalities collaborated with experts-by-experience or other community-partners (Fig. 5). General practitioners (GPs), health insurers and experts-by-experience were most often regarded as missing parties (Table 2).

### Experiences with the implementation of the Solid Start program and cross-sectoral collaboration

A total of 6 FGDs and 19 interviews were conducted, as detailed in Table 3. The findings were outlined in the six dimensions of the RMIC and summarized in Table 4. Table 4 presents an overview of both positive experiences and developments in the implementation of Solid Start and cross-sectoral collaboration, as well as the challenges that remain and the needs for improvement.

**Table 3:** Number of participants in FGD’s and interviews

	Total (unique)^a^	2019	2020	2021
Representatives of care and support organizations (both managers and healthcare providers)	14	7	4	4 (incl. 1 individual interview)
- Social sector	- 7	- 5	- 2	- 0
- Medical sector	- 7	- 2	- 2	- 4
Solid Start project leaders or advisors	18	6	4	12 (8 individual interviews and 2 interviews with 2 respondents from the same coalition)
Other municipal officials	4	2	2	NA
Researchers and representatives of national knowledge institutes and professional associations	18	6	8	10 (incl. 1 individual interview)
- Social sector	- 9	- 4	- 3	- 4
- Medical sector	- 9	- 2	- 5	- 6
Clients and experts-by-experience	7	0	0	7 (all individual interviews)
**Data collection**	6 FGD’s (2 live, 4 online); 19 interviews (4 live, 15 online)	2 FGD’s (live)	2 FGD’s (online)	2 FGD’s (online); 19 interviews (with 1 or 2 respondents; 4 live, 15 online)

^a^Some stakeholders participated in 2 or 3 rounds

**Table 4:** Overview of qualitative findings

**Dimensions**	**Positive experiences and recent developments**	**Challenges ahead and needs for improvement**
Normative integration	- Increased sense of urgency of importance first thousand days - Increased mutual acquaintanceship (knowing each other) - Visionary leaders facilitated Solid Start (e.g. national advocates and local ‘coalition of the willing’)	- Further transcending domain perceptions and overcoming cultural differences and fragmented structures
Professional integration	- Agreements on interdisciplinary collaboration have increased - Multidisciplinary guidelines, protocols, interventions and policies for Solid Start have increased - Shared goal to provide every child a solid start stimulated collaboration - Experiencing value creation (‘what’s in it for me as a professional’) stimulated collaboration	- Successful implementation of agreements, guidelines, protocols, interventions and policies in practice - Integration of Solid Start into all professionals’ daily practice
Organizational integration	- Centering the needs of the target population as binding agent stimulated collaboration - Dedicated initiators or project leaders were a driving force - Increased learning and knowledge sharing - Increased monitoring and evaluation - Learning community to support the setup of local monitoring - Experiencing value creation (‘what’s in it for the organization’) stimulated collaboration - Support from (executive) board members and aldermen	- Continuation of driving forces at institutional level - Challenges related to organizational features - Complexity in one sector hinders cross-sector collaboration - Challenges in monitoring like data-availability, selecting relevant indicators, correct interpretation - Continuing learning between and within Solid Start coalitions - Learning from sectors other than Solid Start (e.g. elderly care) and disseminating knowledge based on Solid Start experiences to other sectors
Clinical integration	- Increased attention for continuity of care, case management and client-centered care - Improved interpersonal interaction between clients and professionals - Increased client involvement in the organization of care - Increased client involvement in daily practice (shared decision-making) - Learning programs to support client involvement	- Further shifting from supply-oriented care and support to prioritizing clients’ needs—Improving interpersonal interaction - Standardizing client involvement - More involvement of partner/spouse and wider informal network - Better focus to clients’ experiences and satisfaction for improvements - Improving the completeness, diversity and communication of client-information to enhance alignment and identification
Functional integration	- Support for coalition building and implementation of Solid Start activities at local level	- Integrated information system to share information between professionals
System integration	- Solid Start funds facilitated implementation on local level - Increased involvement stakeholders from social and medical sector - The Solid Start program’s structure was appreciated for its governmental stewardship and strong local focus - Solid Start creates social value at system level - Previous local cross-sectoral projects targeted at health and disparities (during pregnancy) were used as starting point	- Ensuring structural and sustainable funds for long-term planning - Involving more GPs, health insurers, experts-by-experience - Adapting the scope of laws and regulations to stimulate Solid Start, with regard to cross-sectoral collaboration and task-division - Aligning integration with coalitions’ and professionals’ needs for support - Facilitating knowledge development and dissemination - Acknowledging Solid Start as ultimate form of prevention - More insight into impact, cost-effectiveness and success factors to maintain its prioritization and political support at local level - Solid Start is regarded as a transition rather than an innovation; transitions are complex and time-consuming - Balancing workload, limited time and capacity (workforce shortage) with adequate care and support is challenging - COVID-19 pandemic decreased funds, manpower and priorities for Solid Start

In the next sections, we explain the key results, provide examples and highlight the interconnections between different levels of the RMIC. The order of the dimensions was determined by the stakeholder’s narratives. As normative integration seemed to be a fundamental step towards more integration in relation to Solid Start, this dimension was positioned at the top of the table and discussed first.

#### Normative integration

The experiences of stakeholders seemed to reveal that normative integration was the starting point for more collaboration and integration in relation to Solid Start. During almost all conversations, stakeholders stressed how Solid Start created more sense of urgency regarding the importance of the first thousand days and prevention, and feelings of collective responsibility to coordinate care and support for parents and children. This increased sense of urgency had implications at different levels (micro, meso and macro) and was a starting point to initiate or intensify activities. However, especially in 2019, stakeholders also described difficulties in deciding where and how to begin with the local implementation of Solid Start. Most municipalities started building their coalition by organizing a kick-off meeting with relevant parties to discuss current workflows, challenges and strengths. These and other meetings contributed to mutual acquaintanceship between individuals from different organizations, as they got to know each other and gained insight in each other’s expertise. This led to quick gains such as the exchange of contact details and casuistry, and long-term benefits such as increased trust, understanding, learning and contact for future clients. This quote of a participant in a FGD illustrates how getting to know each other can improve the collaboration:

*“And I think, the moment we know about each other, what each other's expertise is, what you're good at, how you can support the other, that's already very helpful to be able to start forming a local coalition and to start organizing care together around vulnerable pregnant women.”*—FGD, 2020

Stakeholders described how the historical separation and fragmentation between the medical and social sector was persistent and challenging to overcome. Involved organizations often had different cultures, languages, ways of working, legislations, structures, focus areas, networks and missions, which were frequently mentioned as barriers to collaboration. Practical examples included differences in working hours that impeded finding a time to meet. Other examples included a difference between working supply-oriented or demand-oriented, curative versus preventive, focused on children versus parents, and no common understanding of vulnerability. Stakeholders expressed a need for providers to move beyond their own professional perspectives, to further transcend domain perceptions, and overcome cultural differences and fragmented structures. Besides getting to know each other, elements such as developing a shared vision and objectives, and joint multidisciplinary education were considered as helpful.

#### Professional integration

At the professional level, stakeholders reported more agreements on interdisciplinary collaboration. Over the past years, there has been an increase in multidisciplinary guidelines, protocols, interventions and policies for the first thousand days. For example, multiple stakeholders reported the initiation or expansion of multidisciplinary meetings and joint intakes. Moreover, there has been an increase in the use of structured risk screening tools that focused on both medical and social factors. Additionally, tailored multidisciplinary care pathways for vulnerable pregnancies have been developed or refined to ensure timely and appropriate referral. However, the agreements made did not ensure successful implementation in practice, due to several reasons. For example, the high number of professionals made it difficult to get everyone together, and sometimes there was sufficient funding to develop guidelines but not enough to implement them, despite a stakeholder’s view that *“implementation remains most important, regardless of all the documents”* (FGD, 2021). Implementation in practice was considered an ongoing challenge and stakeholders wished for greater alignment in processes in the coming years.

Additionally, notwithstanding numerous developments at the professional level, the Solid Start program and the need for collaboration are not yet fully incorporated into all professionals’ everyday practice. Stakeholders have emphasized the need for everyone to acknowledge its importance and take responsibility. As one stakeholder stated: *“There is a need for change, there is potential for change, if we do it together.”* (FGD, 2021) Several stakeholders agreed that this can be stimulated by including Solid Start in curricula and professional profiles. Moreover, professionals must receive practical tools, adequate support, and training to enhance their competences. These competences include, but are not limited to, effective communication and interacting with clients in a cultural and stress sensitive way.

#### Organizational integration

The Solid Start program enabled organizational integration by centering the needs and preferences of the target population as a binding agent at the core of all activities. One of the stakeholders explained this by noting: *“What the added value is, is the focus on the child. And not just on disciplines or sectors, domains and acquired practices.”* (FGD, 2020). Moreover, a dedicated local initiator, project leader or coordinator as driving force was considered essential for coalitions’ progress. Someone who brings parties together, facilitates and takes an ambassadorial role. Despite differences, this position was often filled by someone from public health services, a regional support structure, the municipality, or another neutral party. Stakeholders provided examples where the development halted when that person left. Therefore, they suggested that these driving forces should be institutionalized and supported financially and practically in the future.

Several challenges that arose at the organizational level were related to different organizational features. For example, municipalities and care and support organizations had different structures and their physical working areas often did not completely overlap. The social sector was described as fragmented, in contrast to birth care in which organizations often united in obstetric partnerships. Additionally, several organizations, including youth healthcare (preventive public health services to promote health and development for children from birth till the age of eighteen), work in multiple municipalities. The differences and fragmentation made it harder to reach agreements between organizations. Stakeholders also mentioned how the perceived difficulties arising from developments within one organization or sector (e.g. integrated birth care and transitions in youth care) could complicate cross-sectoral collaboration for Solid Start as well.

Learning and sharing knowledge were frequently mentioned as essential to improve integration. Stakeholders highlighted how the existence and design of Solid Start fostered learning opportunities. Municipalities sought to learn from best practices in other municipalities in order to avoid unnecessary duplication of efforts. They did so for topics ranging from ‘developing a local approach with a comprehensive set of interventions’ in 2019 to ‘monitoring and ensuring/embedding the approach’ in later years. One of the stakeholders mentioned: *“[…] we also keep a close watch on what other regions are doing, what can we learn from them?”* (FGD, 2020). As such, stakeholders emphasized the importance of learning and knowledge sharing in the future, both between and within coalitions.

The qualitative data showed that municipalities started with monitoring and evaluation. Examples were provided about discussing data and indicators on processes and outcomes during the first thousand days with professionals at municipal or neighborhood-level, in order to understand local developments and prioritize future actions. However, many municipalities had questions regarding monitoring, such as: which indicators to include, how to start monitoring, where to find data and how to interpret the data? Support from RIVM’s learning community to aid the setup of local monitoring was appreciated.

#### Clinical integration

Primarily clients, experts-by-experience and professionals have reported on the concepts of continuity of care, case management and client-centered care. These concepts, which prioritize the central role of clients’ needs, have gained increasing attention in recent years and have come to influence the provision of care and support. For example, several local coalitions engaged in discussions on how (future) parents navigate care and support provided during the first thousand days to uncover areas for improvement. Despite progress, stakeholders mentioned that care and support were still too much driven by policy and professionals (supply-oriented) and prioritizing clients’ needs was not yet routine practice. Stakeholders expressed the need to better address the multiple challenges faced by (future) parents in vulnerable situations (e.g. related to housing, work, education and parenting). This requires restructuring and improved communication among the professionals involved. In some areas, case managers were appointed or central telephone numbers for referrals have been implemented. One of the clients described her experiences with having one case manager:

*"I had one person I could share everything with, so that was very nice. [...] [she had] conversations with me about how I feel, but also about finances." *- Client, 2021

Stakeholders also reported that although improvements have been made in the interpersonal interaction between professionals and clients, there remains a need for further development. Clients and experts-by-experience shared both positive and negative experiences. Positive experiences were associated with the keywords empathy, understanding, respect, transparency, safety, trust, and being heard and understood. Negative experiences, however, were marked by incidents of prejudice, judgement and underestimation. To enhance interpersonal interaction, stakeholders have emphasized the need for training in sensitivity and communication. Everyone is different and “[…] *to me this means that you really look at the person and the situation.*” (Expert-by-experience, 2021).

Lastly, stakeholders have noted increased client involvement, both in the organization of care and in daily practice. For example, several organizations established parent or client councils, and the Ministry of VWS invited a group of experts-by-experience to reflect on national policy measures since mid-2020. However, stakeholders also mentioned the need to expand and standardize client involvement for quality improvement. They mentioned challenges including how to start and involve the right people, and emphasized that it is important to consider financial reimbursements. Mainly since 2021, client involvement became a more central topic for coalitions and Pharos started to organize learning programs to support this effort. In daily practice, shared decision making and positive health principles supported client involvement, allowing for putting parents’ needs and preferences first in decisions concerning their own health and well-being.

#### Functional integration

Pharos has supported municipalities since 2019 in building their coalition, which was highly valued. Municipalities had varying needs for support, depending on the coalitions’ developmental stage and other factors. The need for one-on-one support seemed to have shifted towards a need for mutual knowledge-exchange over time. As previously explained, stakeholders requested additional support for professionals to incorporate Solid Start into everyday practice. A participant in a FGD said:

*“Ultimately, you do it for the children and their parents, but you need to give the professionals tools to be able to continue to do this.” *– FGD, 2021

The FGDs revealed difficulties in sharing information between professionals, particularly in the context of referrals. This was complicated by General Data Protection Regulations according to the stakeholders. Some stakeholders called for an integrated information system and more transparency. Although digital data exchange in birth care has been in development for a few years, it was not yet standard practice.

#### System integration

We have found several systemic determinants that influenced collaboration at meso- and microlevel. Overall, most challenges that arose in the interviews and FGD’s seemed to concern systemic integration. Hence, stakeholders highlighted a range of needs that should be addressed in order to embed Solid Start and ensure the program’s sustainability. One of the stakeholders explained her view, which was supported by many others:

*"It is really a transition from the system as it was, you know, quite a fragmented system. […] Even four years is very short for that, right? So you're really setting a movement in motion, and I think that program is really setting that in motion. But it is really a long-term issue, simply because you are changing a lot of things. [...] When you really want to get it into the system, and therefore want to see improvements in collaboration everywhere, then these annoying prerequisites come up again, right? Then you have to make sure that financing follows as well, that it supports care instead of getting in the way, for example. Those kind of things.*” - FGD, 2021

In relation to available resources, stakeholders mentioned that the Solid Start funds helped to start activities at local level. However, the funds were frequently described as limited, temporary and project-oriented, thereby impeding long-term planning. Municipalities reported difficulties to obtain funds for interventions, and to bring partners together without reimbursements. Stakeholders noted that funds were often invested in innovation and curation rather than implementation and prevention. Moreover, they generally mentioned unclarity regarding prevention. For various preventive activities related to Solid Start, it was unclear to the stakeholders whether the municipality or health insurers should bear the financial responsibility, resulting in occasions where funds were unavailable. There are five different Dutch laws that include prevention, which complicated the financing and funding thereof. Another difficulty was that investing in preventive measures can be uncertain and may not always benefit the investor (wrong pocket issue). Over the years, but peaking in 2021, stakeholders have called for structural and sustainable funding to ensure Solid Start’s sustainability.

Next, stakeholders noted increased involvement of organizations and professionals from the medical and social sector. The composition of coalitions varied based on factors such as the municipalities’ focus, challenges and historical context. General practitioners, health insurers and experts-by-experience were mentioned as major missing parties. Stakeholders anticipated that GPs, who are potentially vital in preconception care, were often unavailable due to their heavy workload and because they did not view Solid Start as a core activity. Health insurers were seen as a potential source of funding for preventive activities, although discussions about this were experienced as difficult due to the health insurers’ focus on individuals (indicated prevention) rather than on groups (universal or selective prevention).

Moreover, stakeholders mentioned several laws and regulations that hindered cross-sectoral collaboration. One example concerned the legal task of youth healthcare to enhance children’s health and development (0 – 18 years), which lacks a focus on pregnancy and (future) parents. At the time of data-collection, a law was being prepared that gave municipalities the responsibility to implement prenatal home visits by youth healthcare. This expands the scope of youth healthcare and was well-received. Another example was the ambiguity of midwives’ role in promoting preconception health, as they usually meet expectant mothers during pregnancy. Several stakeholders called for better preconception care arrangements. Lastly, when other crises were perceived as more immediate (e.g. COVID-19 pandemic for Public Health Services), organizations tend to focus on their core activities written in laws and regulations, which may not always include Solid Start. Hence, stakeholders expressed a need to adapt the scope of laws and regulations to facilitate Solid Start. Additionally, they mentioned that well-defined procedures, roles and responsibilities could help to eliminate a lack of commitment. They suggested for example that an organizational entity should be allocated with the responsibility to serve as the driving force to continue with Solid Start, even if funding by the Ministry of VWS would stop.

Stakeholders appreciated the national Solid Start program’s design and structure, which features national governmental stewardship and a strong local focus and infrastructure. They acknowledged that the program's emphasis on local considerations was appropriate, given the unique contextual and societal challenges faced by different municipalities. The program provided sufficient autonomy to implement locally without following a rigid, prescriptive checklist. However, stakeholders also sought to ensure the institutionalization and long-term integration of Solid Start and its interventions. Municipalities reported difficulties in moving out of the innovation- and pilot-phase. Stakeholders emphasized, especially in 2021, that they considered Solid Start a ‘transition’ or ‘movement’ rather than a short-term project. Although progress was being made, stakeholders recognized that the program's shift from managerial, policy and executive board levels to individual professionals in daily practice takes time and effort:

*"And we really still need to take the step towards the individual care provider who should work with it, because they are actually in direct contact with that family. [...] I think that’s maybe the most difficult thing, that it doesn’t just stay on those governance tables, but that it’s now transported to where it really needs to be." - * FGD, 2021

In this process, stakeholders suggested to focus on coalitions and professionals’ needs for guidance and support, and to further facilitate knowledge development and dissemination. One of the stakeholders proposed an increase in interactions between national and regional/local level to facilitate these objectives.

Lastly, stakeholders commented that Solid Start should be considered in a wider societal perspective as the ultimate form of prevention to address health disparities and tackle poverty issues. This means that Solid Start should maintain its prioritization. Currently, the system is not entirely in alignment with the overarching mission. The underlying reasons for initiating Solid Start are deeply rooted, complicated and not easily resolved, which was why the stakeholders emphasized that a continuous focus is necessary:

*"I am incredibly happy with a program like Solid Start, because you can just work with many more people and many more municipalities, and extract the effective elements. [..] But if the Solid Start program only lasts four or five years, we haven't tackled the problem, we've just become more aware, and hopefully we've been able to find each other better and hopefully there are people in many municipalities who want to continue being a driving force, but we haven't solved the problem. And we have to get rid of that illusion [that we solve it in four of five years] somehow." *– FGD, 2020

## Discussion

This study aimed to describe the implementation of the Dutch Solid Start program during 2019—2021. Questionnaires, interviews and FGD’s revealed progress in cross-sectoral collaboration over the years, with a growing number of municipalities forming Solid Start coalitions involving diverse stakeholders. Coalition development varied due to municipalities’ unique challenges, focus and historical contexts. According to the stakeholders, initiating the Solid Start program increased the sense of urgency for the first thousand days and stimulated professionals from various backgrounds to get to know each other, resulting in more collaborative agreements on care provision. Stakeholders appreciated the program’s local focus and opportunities for learning. However, they experienced that Solid Start is not yet fully incorporated into all professionals’ everyday practice. Most common barriers related to systemic integration at macro-level, including limited resources and collaboration-impeding regulations. Stakeholders emphasized the importance of ensuring Solid Start’s sustainability.

Our findings suggest that the Solid Start program contributes to the shift from traditional, fragmented care towards a more integrated, population health-based care system as described in literature [39]. This approach involves an increased focus on prevention, recognition of the social determinants of health and improving equity in health and wellbeing [39]. In line with literature about complex persistent problems, societal transitions, system changes and transformations [40–43], stakeholders mentioned that these developments take time and effort. Historically grown specializations and demarcations that once facilitated progress in healthcare now pose significant integration barriers due to separated cultures, structures and legislations. Nevertheless, it seems that Solid Start has created a nationwide movement to integrate medical and social services for early life within a relative short time (mid-2018 till 2021), with modest funding (€41 million allocated throughout the program’s duration) [15, 44] and without a system reform or refiguration. According to Barsties et al.’s transition research in Dutch obstetric care [8], social obstetrics is a new way of thinking, doing and organizing that challenges the incumbent regime that may provide a sustainable addition to the current system, instead of a disruptive transformation. The authors note that social obstetrics can be a starting point for further transformations in obstetrics and other societal systems. Several experts also suggest that systemic structures (e.g. financial structures, laws and regulations) must ultimately transform to achieve greater sustainability and long-term impact than possible through improvements within the current system [43, 45]. The trajectory of such transformational processes is often unpredictable and nonlinear [46]. Our findings reveal various practical and systemic barriers that impede stakeholder efforts to effect change, calling for systemic transformations as well. The path towards improvements in early life will be influenced by political decisions made in the Netherlands.

In any case, stakeholders emphasized the importance to institutionalize Solid Start and ensure the program’s sustainability, to guarantee that the incremental changes result in lasting improvements. Drawing on stakeholders’ perspectives and previous literature, several factors can accelerate this transition. The first factor is structural and sustainable funding. Short-term grants should be considered a bridge towards stable financial arrangements for long-term integration and value-creation [45, 47]. Meanwhile, sustainable arrangements with municipalities, healthcare insurers, and others should be considered to fund prevention and health promotion, which may require local experiments and legal enforcements. The second factor is adapting the scope of laws and regulations to facilitate Solid Start and cross-sectoral collaboration. The recent changes to the Public Health Act since July 1, 2022, for example, require municipalities to provide prenatal home visits by youth healthcare to parents-to-be in vulnerable situations. Stakeholders have requested additional changes, such as legally outlining preconception care and early detection of vulnerability. If such activities are regarded as core tasks due to laws and regulations, organizations and professionals may be less likely to drop Solid Start activities during crises such as COVID-19 and (expected) labor shortages. The need fits the wider call in the Netherlands to embed public health benchmarks in legislation to increase accountability, similar to environmental legislation [48].

Stakeholders have expressed other needs, which concern responsiveness to stakeholders’ needs, ongoing knowledge development, and client-centered care. Firstly, an increased focus to coalitions and professionals’ needs is required, as policy recommendations often fail to be implemented in practice without adequate support [49]. Further developed partnerships require different types of support compared to those in early stages [45, 47]. Additionally, professionals must be supported in adapting to their changing roles and responsibilities in daily practice, as behavioral change is difficult and influenced by multiple factors, including knowledge and skills development [50–52]. Secondly, ongoing knowledge development and dissemination are vital to overcome collaborative challenges and stimulate learning. Many systemic barriers cannot be resolved by individual parties at local level and require changes at higher levels. More interaction between local, regional and national levels through intermediary partners, platforms or boundary spanners may help to create learning opportunities at all levels and to adequately collect and respond to different needs [21]. An example is the RIVM’s local monitoring support program: various coalitions regularly discuss local challenges and successes for mutual learning, and pressing issues are shared with the Ministry of VWS to inform the policy agenda. Thirdly, stakeholders emphasized the importance of putting clients’ experiences and needs central in daily care and its organization. Although there has been progress, stakeholders felt that this required improvement. Client-centered care and participation (in decision-making) can improve the professional-client relationship, increase satisfaction and promote sustainable innovations by considering clients’ preferences, needs, strengths and weaknesses [53, 54].

Our findings are in line with the needs and learning points described in both national and international papers on integrated care and cross-sectoral collaboration in other fields [41, 45, 55–57]. For example, these papers also reported on the importance of interpersonal contact and mutual recognition of each other’s roles and expertise, engaging all stakeholders (including clients), ensuring sustainable finances, fostering learning cycles, adapting to new roles and skills, and having good governance and leadership throughout all levels of the system. Additionally, we identified comparable obstacles to collaborative efforts as documented within the medical maternity care sector such as fragmented structures and cultures, limited resources and impeding regulations [19–26, 30]. Nevertheless, collaborating between sectors seemed to pose additional challenges, given the greater disparities in relational and organizational aspects. For example, the differences between municipal structures and the healthcare system required more investment to foster mutual understanding and familiarity with each other's work environments and interests. Moreover, the financial system was more compartmentalized and governed by distinct regulatory frameworks, presenting challenges in financing preventive measures that are at the intersection of different laws. Lastly, we found that the perceived difficulties from developments within one sector (e.g. integrated birth care, youth care transitions) can complicate cross-sectoral collaboration.

In 2022, the Ministry of VWS published the follow-up approach Solid Start 2022–2025 *Strong parents, healthy children!,* which aspires to create a structural Solid Start approach in every municipality [58, 59]. The approach aligns with the needs expressed in our study. There is a continuous focus on cross-sectoral collaboration at local and regional level, and extra focus to client involvement, facilitating professionals and strengthening informal networks. The approach outlines a commitment to sustainable funding, supportive regulations, governance agreements, a learning infrastructure, monitoring and retain a sense of urgency. Some specific actions have been defined to attain these intentions, while others will be developed. The follow-up approach highlights embedding Solid Start in wider prevention policies and linking it with other policy themes (e.g. poverty) to ensure its sustainability. Given that changes can take decades or span generations [40], during which leadership and contextual circumstances will inevitably change, we need long-term plans beyond the time horizons of a few years to reduce inequities and improve health and well-being [45, 60, 61].

This study offers relevant insights to future policy developments and collaborative practices, and contributes to the knowledge base on cross-sectoral collaboration. Multiple other countries started programs to reduce health inequities by stimulating cross-sectoral collaboration in early life. Examples are the First 1000 days-program in Massachusetts (US) [11], Sure Start in England [12], Strong Start and Healthy Start in the US [13, 62], Strong Start in Australia [14] and Germany’s Early Childhood Intervention program [63]. Future research should synthesize learning points from successes and failures across these programs and countries. Monitoring processes and outcomes on an ongoing basis can support learning for continuous improvements, consistent with the concepts of reflexivity and reflexive monitoring [49, 64, 65]. The importance of monitoring applies to both national and local (municipality) level [66]. Future research should also focus on the effects of Solid Start on health outcomes and utilization.

### Strengths and limitations

Strengths of this study were the extensive data-collection over multiple years and the inclusion of a wide mix of stakeholders, including clients and experts-by-experience. Our data collection seemed to have reached saturation. However, the perspectives of some important parties such as GPs, health insurers and councilors were missed and could have given additional insights. Also, municipalities that did not request Solid Start funds responded less to questionnaires, and we may have involved a selective group of more active and motivated stakeholders in interviews and FGD’s. This may have led to more positive findings, although we noticed that our approach provided a good understanding of barriers to implementation at various levels as well. The approach in which we combined FGDs, interviews and questionnaires contributed to the credibility of our results [67]. Quantitative data increased our understanding of Solid Start implementation nationwide, and qualitative data provided detailed, contextualized insights.

Using the RMIC as analytical framework for our qualitative data was considered useful to better understand collaboration across professionals, organizations, levels and sectors. The RMIC is one of the theoretical models and definitions on collaboration, integrated care and Population Health Management that sought to outline its important elements (e.g. [31, 56, 68, 69]). The model is well able to capture cross-sectoral collaboration. However, as with any other model, the RMIC’s reliance on predefined domains and elements may overlook the complexity and variability of integrated care initiatives in practice. Nonetheless, the multilevel and multidimensional RMIC has a strong theoretical and empirical foundation, as it is based on extensive literature review [31, 38] and widely used in research, also in Dutch maternity care [70]. For this study, using the model has provided greater insight into the significance of normative integration as a primary step in cross-sectoral collaboration, the dynamics among different layers, and the potential for improvement even in the presence of systemic-level barriers that should be addressed over time. In future endeavors, it may be valuable to explore the underlying cognitive processes influencing the implementation of the Solid Start program, for example as outlined by the normalization process theory [71].

## Conclusion

This study shows that the Dutch Solid Start program has created a movement towards a more integrated and population health-based care and support system. Solid Start, as a national program with strong local focus, has led to various incremental changes that supported cross-sectoral collaboration for early life, without major transformations of systemic structures. This study highlights several barriers and needs to address in order to ensure the program’s sustainability. Those include sustainable funding, supportive regulations, responsiveness to professionals’ and coalitions’ needs, ongoing knowledge development, and client involvement. In the near future, it is essential to monitor whether the follow-up approach effectively addresses the barriers and needs.

### Supplementary Information

**Additional file 1: Appendix 1.** Description of the Dutch care and support system during the first thousand days.

**Additional file 2: Appendix 2.** Description of national and local Solid Start monitor by the National Institute for Public Health and the Environment.

## Data Availability

The datasets generated and analyzed during the current study are not publicly available due to the restrictions claimed in the information to respondents and to ensure the protection of anonymity of the participants. Any templates used for data collection and analysis are available from the corresponding author on reasonable request.

## Abbreviations

FGD: Focus Group Discussion
GP: General Practitioner
Ministry of VWS: Dutch Ministry of Health, Welfare and Sport
RIVM: Dutch National Institute for Public Health and the Environment
RMIC: Rainbow Model of Integrated Care
