# Supplementary material for: The Dutch Solid Start program: describing the implementation and experiences of the program's first thousand days
Source: BMC Health Serv Res. 2023 Aug 30;23:926. doi: 10.1186/s12913-023-09873-y (PMC10470180; doi:10.1186/s12913-023-09873-y)
Supplement: Supplementary file 1 — Additional file 1: Appendix 1. Description of the Dutch care and support system during the first thousand days. [file 12913_2023_9873_MOESM1_ESM.docx]

Appendix 1. Description of the Dutch care and support system during the first thousand days

In the Netherlands, (future) parents and children generally receive care and support from different service providers, depending on the (expected) health risks and need for support. During pregnancy, women without medical risk factors are generally seen by primary care midwives and they can choose to give birth at home or in an outpatient clinic. In case of increased medical risks or complications, women are referred to general hospitals (secondary care), or, in case of severe morbidity, to academic hospitals (tertiary care).(1) Obstetricians, hospital-based midwives, obstetric nurses, and pediatricians provide care in the hospital. After birth, maternity care assistants provide postnatal care to mother and baby at home or in a maternity hotel. Children receive youth healthcare services by youth doctors, youth nurses and assistants till the age of 18. Youth healthcare services also provide prenatal home visits to pregnant women and families in a vulnerable situation, following a change in the Public Health Act in July 2022 as part of the Solid Start program.(2, 3) Furthermore, depending on the (future) parents’ circumstances and need for support, they can be referred to service providers or organizations in the social domain or youth care. For example, this could be a municipal housing official for help related to housing, a dept counsellor for support with financial issues, or a social worker or Safe Home (in Dutch: Veilig Thuis, the national report center for domestic violence and child abuse) to intervene in cases of domestic violence.(4, 5) Within each municipality, the type of support that is available to parents can differ. Social support services are paid by municipalities under the Social Support Act (In Dutch: Wet Maatschappelijke Ondersteuning, Wmo) and medical services are reimbursed, mostly on a fee-for-service base, through health insurance companies under the Healthcare Insurance Act (In Dutch: Zorgverzekeringswet, Zvw).(4)

**References**

1. Amelink-Verburg MP, Buitendijk SE. Pregnancy and labour in the Dutch maternity care system: what is normal? The role division between midwives and obstetricians. Journal of midwifery & women's health. 2010;55(3):216-25.

2. Nederlands Centrum Jeugdgezondheid. Handreiking prenataal huisbezoek door de JGZ. Voor zwangeren en/of gezinnen in een kwetsbare situatie. [Guide for prenatal home visits by Youth Health Care (JGZ) for pregnant women and/or families in a vulnerable situation]. Utrecht; 2022.

3. Ministerie van Volksgezondheid, Welzijn en Sport. Wijziging van de Wet publieke gezondheid vanwege het opnemen daarin van een gemeentelijke taak om prenataal huisbezoek te verrichten (Kamerstuk 35593). [Amendment of the Public Health Act to include a municipal task of conducting prenatal home visits]. 2020.

4. Kroneman M, Boerma W, van den Berg M, Groenewegen P, de Jong J, van Ginneken E, et al. Netherlands: health system review. 2016.

5. Schreiber L. A solid start for every child: the Netherlands integrates medical and social care, 2009 - 2022. Princeton University; 2022.
