# Supplementary material for: The Dutch Solid Start program: describing the implementation and experiences of the program's first thousand days
Source: BMC Health Serv Res. 2023 Aug 30;23:926. doi: 10.1186/s12913-023-09873-y (PMC10470180; doi:10.1186/s12913-023-09873-y)
Supplement: Supplementary file 2 — Additional file 2: Appendix 2. Description of national and local Solid Start monitor by the National Institute for Public Health and the Environment. [file 12913_2023_9873_MOESM2_ESM.docx]

Appendix 2. Description of national and local Solid Start monitor by the National Institute for Public Health and the Environment

The Ministry of Health, Welfare and Sport (Dutch abbreviation: VWS) commissioned the National Institute for Public Health and the Environment (Dutch abbreviation: RIVM) to monitor the implementation of the Solid Start program, launched in September 2018. The RIVM began monitoring the program at the national level in 2019 and launched a support program for municipalities called the “Learning Local Monitor Solid Start” in 2021.

**National Solid Start monitor**

The national monitor includes both a quantitative and a qualitative component. In a Delphi study conducted in 2019, experts from policy, practice, and research developed a set of 15 quantitative indicators.(1) The indicators reflect both processes (e.g. percentage of municipalities with a local Solid Start coalition) and outcomes (e.g. percentage of children born prematurely or with a low birth weight). Annual factsheets (2-4) report the figures for each indicator to monitor the program's progress and developments/trends in health outcomes. The RIVM uses several data-sources to quantify the indicators, including:

1. Data from the nationwide population-based data-infrastructure DIAPER (acronym for Data-InfrAstructure for ParEnts and ChildRen). DIAPER integrates routinely collected observational data from three Dutch nationwide data sources (Perined, Vektis and Statistics Netherlands) at individual level. *Perined* is the national pregnancy, birth and neonatal data registry, based on data supplied by midwives, obstetricians and paediatricians. *Vektis* offers data on healthcare utilization and spending by collecting claims data under the Dutch Healthcare Insurance Act. *Statistics Netherlands* collects, disseminates and facilitates access to data on societal themes, including health, welfare, income, education and labour
2. Inquiries to national organizations involved in the implementation of interventions and youth healthcare organizations, as there is no national youth healthcare data registry in the Netherlands
3. Questionnaires among municipalities^[[1]](#footnote-2)^

The qualitative component involves interviews and focus group discussions with stakeholders that provide further insight into the factors that facilitate or hinder the implementation of the program.(4)^1^

The MoH uses the results of the monitor in combination with other data sources and expert opinions to determine whether goals are being achieved and to timely adjust policies. To underpin the key-messages within the factsheets and to provide a scientific base for our work for Solid Start, in-depth scientific research and analyses are conducted. This manuscript serves as an example.

**Learning Local Monitor Solid Start**

In 2021, the RIVM started providing support to municipalities in setting up local monitoring, as several of them expressed a need for such support. The support program aims to encourage local coalitions to utilize monitoring as a tool to enhance and refine their local approach. Key elements of the program include the establishment of a learning community that fosters mutual learning among stakeholders (both within and between local coalitions) and encourage the sharing of best practices.

Eleven representatives from local coalitions participate in regular learning sessions. They were already engaged in monitoring Solid Start at the local level before or in the early stages of the national program. This group inspires each other by sharing their experiences and best practices. They also discuss challenges and needs for support in local monitoring. Examples include ‘what is vulnerability?’ and ‘how to monitor the collaboration between the medical and social domain?’ These themes are elaborated upon in thematic meetings that are accessible to a broader audience of other municipalities and professionals.

The representatives considered the development of a suitable indicator set the essential first step to stimulate monitoring on a local level. In a previous paper, we have described our approach in developing an indicator set to monitor the Solid Start program in Dutch local coalitions and we presented this indicator set.(5) These local indicators are quantified and presented to all municipalities in the Netherlands at [www.regiobeeld.nl/kansrijke-start](file:///\\alt.rivm.nl\Data4\Projecten\V010038%20Kansrijke%20Start\Promotie%20Joyce%20Molenaar\Artikel%203.%20Kwalitatief%20artikel%20KS\www.regiobeeld.nl\kansrijke-start). In the future, this website will be further refined with additional indicators and new functionalities.

**References**

1. RIVM. Indicatoren Kansrijke Start: Een Delphi-studie [Indicators Solid Start: a Delphi study]. Bilthoven, the Netherlands; 2019. [https://www.rivm.nl/documenten/

indicatoren-kansrijke-start-delphi-studie].

2. RIVM. Monitor Kansrijke Start 2019 [Monitor Solid Start 2019]. Bilthoven, the Netherlands; 2019. [https://www.rivm.nl/documenten/factsheet-kansrijke-start].

3. RIVM. Monitor Kansrijke Start 2020 [Monitor Solid Start 2020]. Bilthoven, the Netherlands; 2020. [https://www.rivm.nl/documenten/monitor-kansrijke-start-2020].

4. RIVM. Monitor Kansrijke Start 2021 [Monitor Solid Start 2021]. Bilthoven, the Netherlands; 2021. [https://www.rivm.nl/documenten/monitor-kansrijke-start-2021].

5. Molenaar JM, Boesveld IC, Kiefte-de Jong JC, Struijs JN. Monitoring the Dutch Solid Start Program: Developing an Indicator Set for Municipalities to Monitor their First Thousand Days-Approach. International Journal of Integrated Care. 2022;22(4).

1. The results of these elements of the monitor are presented in this paper [↑](#footnote-ref-2)
